# Devil in the details: Mechanistic variations impact information transfer across models of transcriptional cascades

**DOI:** 10.1371/journal.pone.0245094

**Published:** 2021-01-13

**Authors:** Michael A. Rowland, Kevin R. Pilkiewicz, Michael L. Mayo

**Affiliations:** Environmental Laboratory, U.S. Army Engineer Research and Development Center, Vicksburg, MS, United States of America; University of Edinburgh, UNITED KINGDOM

## Abstract

The transcriptional network determines a cell’s internal state by regulating protein expression in response to changes in the local environment. Due to the interconnected nature of this network, information encoded in the abundance of various proteins will often propagate across chains of noisy intermediate signaling events. The data-processing inequality (DPI) leads us to expect that this intracellular game of “telephone” should degrade this type of signal, with longer chains losing successively more information to noise. However, a previous modeling effort predicted that because the steps of these signaling cascades do not truly represent independent stages of data processing, the limits of the DPI could seemingly be surpassed, and the amount of transmitted information could actually *increase* with chain length. What that work did not examine was whether this regime of growing information transmission was attainable by a signaling system constrained by the mechanistic details of more complex protein-binding kinetics. Here we address this knowledge gap through the lens of information theory by examining a model that explicitly accounts for the binding of each transcription factor to DNA. We analyze this model by comparing stochastic simulations of the fully nonlinear kinetics to simulations constrained by the linear response approximations that displayed a regime of growing information. Our simulations show that even when molecular binding is considered, there remains a regime wherein the transmitted information can grow with cascade length, but ends after a critical number of links determined by the kinetic parameter values. This inflection point marks where correlations decay in response to an oversaturation of binding sites, screening informative transcription factor fluctuations from further propagation down the chain where they eventually become indistinguishable from the surrounding levels of noise.

## Introduction

Studies over the past half century have made it clear that eukaryotic gene-regulatory networks are exceedingly complex. Within these networks, proteins appropriately named transcription factors (TFs) bind to regulatory elements within promoter regions of DNA to modulate the transcriptional rates of genes [1]. Once TFs bind to DNA, they may recruit other elements to activate the transcription of the gene or act to block additional critical binding events needed for transcription [1, 2]. TFs are able to bind multiple sites, although with varying levels of specificity, and some genes require interactions with multiple TFs to initiate transcription [2–5]. In the *Escherichia coli* bacterium (*E*. *coli*), for instance, the gene regulatory network is hierarchically organized so that only a handful of “global” TFs remain unregulated by any others, with more precise regulation controlled by co-regulation with local TFs [6]. This level of complexity can generate network structures in which a gene is controlled, directly or indirectly, by many upstream TFs. The *E*. *coli* gene *slp*, for example, is regulated by 17 different TFs [7]. This begs an important question regarding control of this and other biologically networked systems: To what extent can gene expression be reliably influenced by fluctuations in the activity of a TF that is several regulatory links removed? In other words, to what extent can the regulatory biology effectively convey an “upstream” signaling event if the information must propagate over a noisy molecular cascade?

The activity level of a TF (e.g., its time-series abundance within the nucleus) directly influences the response of a cell to changes in the environment. Biological functions, however, are inherently noisy, in this case either from the influence of the rest of the gene regulatory network, or through physical noise, such as the impact of Brownian motion on the binding kinetics between a TF and its binding site(s) [8–12]. The ability of a system to identify a signal fluctuation from the pervasive noise, and respond to it appropriately (what we have dubbed the *fluctuation sensitivity* [13]), can be quantified as the mutual information between the input and the output signals, i.e., between the time-dependent fluctuations in the concentration of a TF and those of some directly or indirectly regulated gene product [14]. In previous work, we investigated how the information propagated across a “daisy-chain” cascade of concatenated transcriptional regulatory events varied with the length of the cascade, as well as the linearized kinetic rate constants of the regulatory interactions. We found that, under certain conditions, longer cascades could exhibit higher mutual information than shorter cascades, seemingly in violation of the data-processing inequality (DPI) [13]. No actual violation occurs, however, because the dependence of a gene’s regulation on the steady-state concentrations of all upstream transcription factors ensures that the individual regulatory interactions are statistically dependent upon one another; in other words, the fluctuations in protein abundance at the beginning and ends of a cascade remain significantly correlated despite the presence of noise.

By assuming that the kinetics of transcription were sufficiently well-described by their values near a homeostatic steady state, we linearized the fully nonlinear kinetics and found that protein production should outweigh its destruction to permit growth of information across successive cascade events. What we did not previously consider was whether such a regime was truly feasible in an actual biological system. (At the very least, it could not be sustainable for infinitely long cascades due to fluctuations increasing in magnitude across the chain, which would eventually violate our assumption of small fluctuations at steady state.) In this work, we address this concern by considering a more biologically relevant model of gene regulation that takes into account the explicit binding kinetics of each TF associating and dissociating with sequences within the transcription-initiating regions of DNA. To properly capture the fully nonlinear character of this kinetic model, we simulate it *in silico* using the Stochastic Simulation Algorithm (SSA) [15]. Using these simulation data, we compute the mutual information between fluctuations in the abundance of a “source” TF and the final gene product produced by the terminus of a daisy chain composed of transcriptional-regulatory interactions that we assume rate-limits protein production. Although adequately sampling the probability mass functions underlying this mutual information turns out to be a technical challenge for longer cascades due to the increasing size of fluctuations, we ultimately find that although the fluctuation sensitivity can be enhanced by initially increasing the length of the cascade, these gains are lost as the cascade continues to grow. Applying our linearized theory to this “explicit-binding” model, we find that it grossly overestimates the quantitative value of the mutual information; it nonetheless reproduces the qualitative, nonmonotonic behavior of initial growth followed by rapid decay seen in the mutual information computed from the SSA simulations. Importantly, the theory makes it clear that the eventual quenching of the fluctuation sensitivity occurs as a result of an emergent separation in time scales between the binding kinetics and those of the actual transcription process. This almost adiabatic separation interferes with communication between the steps of the cascade, resulting in the suppression of information about TF molecule fluctuations.

## Results

In our previous work, we used a generalized model for transcriptional kinetics that we linearized for small fluctuations about steady state. Our model of a regulatory cascade was simplified by assuming that the linearized rate constants of each production/destruction process were equal in value across the chain. The most trivial (and least contrived) realization of this regime would be the case where each gene is regulated by an identical mass-action rate law in which temporal changes to the concentration of its encoded protein are directly proportional to the concentration of its regulating TF:

$$\frac{d{\delta R}_{i}}{dt}=k{\delta R}_{i-1}-k_{d}\delta R_{i}+\eta_{i}$$ [1]

In the above, *δR*_*i*_ is the time-dependent concentration fluctuation of the *i*^*th*^ TF in the signaling cascade from its steady-state mean value (in other words, the *i*^*th*^ response to the initiating signal), *k* is the linear rate constant for TF production, and *k*_*d*_ is the rate constant of TF degradation. The function *η*_*i*_ is a delta-correlated Brownian noise term with zero mean, which we use to approximate the stochastic fluctuations caused by all the complex cellular machinery that we neglect to model explicitly. As stated, we assume that rate constants have identical values across the chain, each noise function has an identical statistical distribution, and *R*_0_ is understood to be the concentration of the TF that initiates the cascade. For the sake of achieving closed-form analytic results, we neglected to model the regulation of this lead TF, instead assuming that it remained at a fixed homeostatic concentration until time *t*, at which point it experienced an instantaneous, stochastic fluctuation (the signal) drawn from the same distribution as that characterizing the noise in each other protein concentration. We then used the metric of mutual information to study how this signal correlated with the instantaneous response of each downstream TF population in the cascade. It should be noted that this response can only be instantaneous when the discrete molecular events of transcription are coarse grained as continuum processes, which is a reasonable approximation when studying protein fluctuations across an entire cellular population.

Under the above assumptions, we ultimately found that the condition for the fluctuation sensitivity of the cascade to grow with the number of links was $k>\sqrt{2}k_{d}$. So long as the signal fluctuation is, on average, the same size as a typical noise fluctuation, this result is independent of the noise strength. In this regime, the steady-state concentration of each TF is magnified by a factor *k*/*k*_*d*_ relative to the concentration of its regulator, and the effect of every random concentration fluctuation is similarly magnified across succeeding generations in the cascade. The fluctuations in the source TF always travel at least one more link than any other noisy fluctuation, which means that the signal always gets magnified more than the noise. We also work in the long-time limit (*t*→∞), where the impact of any single noise fluctuation tends to be dampened out over time by countless other fluctuations. By contrast, the signal excitation does not occur until time *t*, so its impact is, by assumption, not attenuated. This ultimately enables the signal-to-noise ratio—and thus the fluctuation sensitivity—to increase with longer cascades.

Clearly, this theoretical framework sacrifices a fair amount of physical realism in exchange for tractable mathematics. Indeed, the very basis for using continuum chemical kinetics to describe the discrete regulatory processes of individual cells relies on an assumption that the dynamics of a large population of cells can effectively be treated as one giant biochemical reactor. Stochastic fluctuations in the concentration of the signal TF would also have to be taken into account in a more realistic treatment, and would surely inhibit the growth potential of the fluctuation sensitivity. Our previous work demonstrated this latter point with some simple stochastic simulations of discretized mass-action kinetics, though a statistically significant growth trend with cascade length was still observed for *k*≫*k*_*d*_. Our objective in this work is to modify those simulations to further relax the cruder assumptions of our analytic model and thereby determine whether information gains across a cascade might be expected in more biologically plausible scenarios. In addition to allowing the number of signaling proteins to fluctuate stochastically, these expanded simulations will include an explicit treatment of the nonlinear protein-binding kinetics central to transcriptional signaling and will be parametrized to describe signaling within a single cell rather than an entire cellular population.

To meet this objective, we develop and study two models in which information encoded by molecular fluctuations propagates via regulatory interactions with differing kinetic mechanisms. In our first model, protein production and destruction rates are linearly dependent upon the concentrations, and its deterministic kinetics can be expressed by the following set of differential rate laws:

$$\frac{dR_{i}}{dt}=kR_{i-1}-k_{d}R_{i}.$$ [2]

We refer to this as the mass-action (MA) model, and note that it is essentially equivalent to **Eq [1]**, except that we have expressed its kinetics in terms of the absolute concentrations in order to emphasize that they are linear by construction, and not by linearization about a steady state. We have likewise suppressed the stochastic component of these kinetics to emphasize that the fluctuations in our simulated models will be controlled by the various reaction rates, rather than being imposed, as in our original analytic model, by a simple Brownian process.

Our second model modifies the kinetic mechanisms of **Eq [2]** as a step toward biological fidelity. If *R*_*i*_ is once again the concentration of the *i*^*th*^ TF in the cascade, *B*_*i*_ is the concentration of free DNA sites that bind that TF, and *R*_*i*_∙*B*_*i*_ is the concentration of those sites that have reversibly bound a TF molecule, then the deterministic component of this second model’s kinetics can be represented by the following set of differential rate laws:

$$\frac{dR_{i}}{dt}=q_{i}R_{i-1}∙B_{i-1}-k_{+}(R_{i})(B_{i})+k_{-}R_{i}{∙B}_{i}-k_{d}R_{i}$$

$$\frac{dR_{i}{∙B}_{i}}{dt}=k_{+}(R_{i})(B_{i})-k_{-}R_{i}{∙B}_{i}.$$ [3]

**Fig 1:**
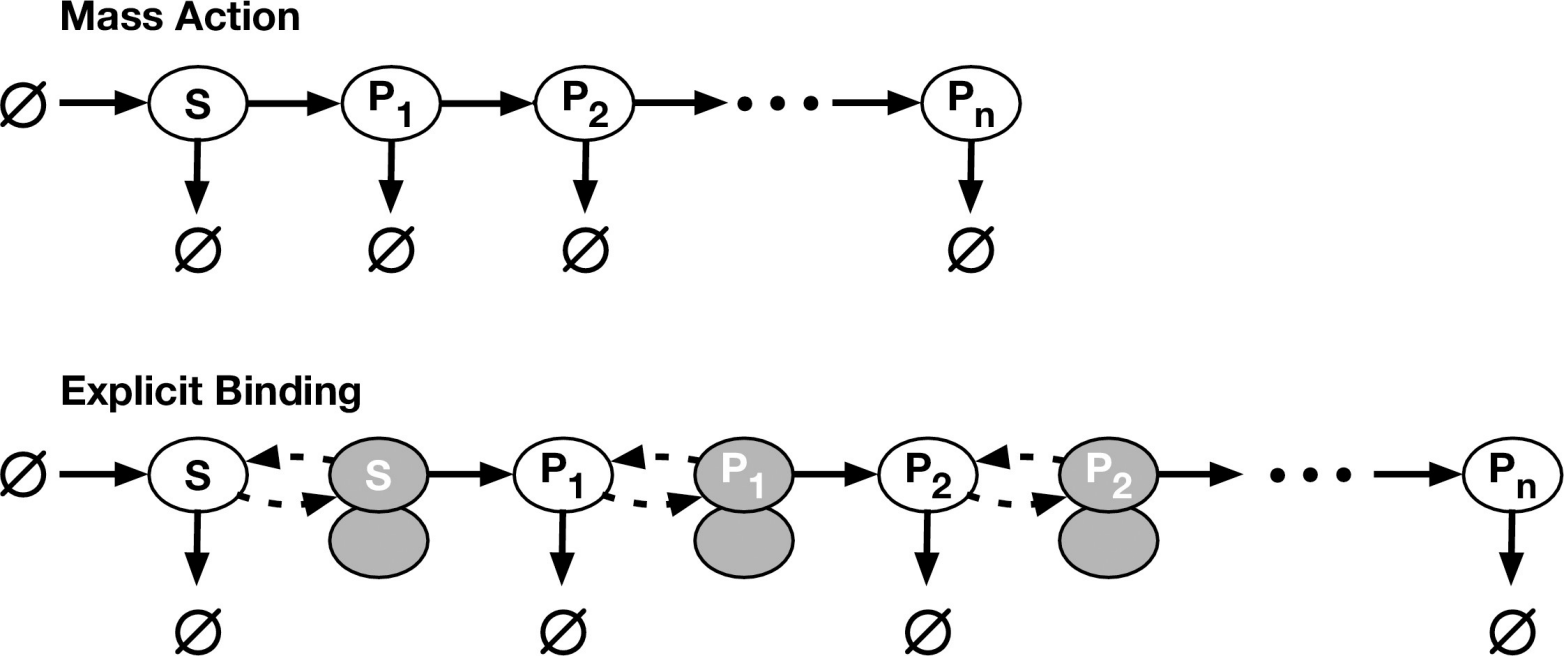
Diagram of the mass-action and explicit-binding models. In the mass-action model, source TF (*S*) is created and destroyed to maintain it at a steady state. *S* regulates the synthesis of gene product *R*_1_, *R*_1_ regulates *R*_2_ (when present), etc. The explicit-binding model is similar to the mass-action, except a TF must first bind to a DNA binding site (bound TFs are shaded in the diagram) before it can stimulate the synthesis of its product. Note that the final product, not being a TF, does not have its own binding site.

We refer to this as the “explicit binding” (EB) model and further assume that kinetics of binding, unbinding, and protein catabolism are identical across the cascade, so that the rate constants *k*_+_, *k*_*−*_, and *k*_*d*_ are of identical value for all TF species. The transcriptional kinetics, which we assume rate-limits protein production, can be different for each link in the chain; however, we shall choose values of *q*_*i*_ that allow for a fair comparison between this model and the simpler mass-action model in the regime where fluctuation sensitivity was predicted to grow with cascade length. **Fig 1** provides a schematic of the elementary reactions that are part of a transcriptional signaling cascade described by **Eq [2]** and **Eq [3].** For the sake of clarity, we once again stress that these two models will be compared through the lens of stochastic simulation. A comparison between the simulation results for each model and the predictions of our previously derived analytic model [13] will be postponed to the end of this section.

In the MA model, the average steady-state concentrations of the various species are related to one another by the following recursion:

$$\langle R_{i,0}\rangle =\frac{k}{k_{d}}\langle R_{i-1,0}\rangle ,$$ [4]

wherein the angled brackets, 〈∙〉, denote temporal averages, and we have defined *R*_*i*,0_ as the *total* concentration of the *i*^*th*^ TF protein. In the mass-action model, *R*_*i*,0_ = *R*_*i*_, but in the explicit-binding model *R*_*i*,0_ = *R*_*i*_+*R*_*i*_∙*B*_*i*_. To make a fair comparison between the two models, we want their kinetics to both fluctuate about the same set of steady-state concentrations {〈*R*_*i*,0_〉, ∀i}. If, as we have assumed, transcription initiation is rate-limiting, then we can leverage the resulting timescale separation to approximately treat the concentration *R*_*i*_∙*B*_*i*_ as if it were always at a steady state. This Briggs-Haldane quasi-steady state assumption (QSSA) amounts to an adiabatic separation of the frequent binding and unbinding kinetics and the slow, irreversible kinetics of transcription itself. Applying the QSSA to **Eq [3]**, we can derive the following pair of equations:

$$q_{i}=\frac{k_{d}\langle R_{i}\rangle }{\langle R_{i-1,0}\rangle -\langle R_{i-1}\rangle }$$

$$\langle R_{i}\rangle =\frac{1}{2}\left[\langle R_{i,0}\rangle -B_{i,0}-K_{D}+\sqrt{{\left(\langle R_{i,0}\rangle -B_{i,0}-K_{D}\right)}^{2}+{4K}_{D}\langle R_{i,0}\rangle }\right]$$ [5]

which, when combined with **Eq [3]**, can be solved iteratively to select the values of the *q*_*i*_ required to restrict both models to identical mean steady states. Note that in the above, we have defined *B*_*i*,0_≡*B*_*i*_+*R*_*i*_∙*B*_*i*_ as the total number of binding sites for the *i*^*th*^ TF, which we assume to be fixed since these sites can neither be created nor destroyed, and we have introduced the dissociation constant *K*_*D*_ ≡*k*_−_/*k*_+_. When recursively solving for the rate constants *q*_*i*_, we assume that 〈*R*_0,0_〉 and *B*_*i*,0_ are known for all *i* and are inputs of the model.

In general, the average concentrations calculated from the chemical master equation do not quantitatively agree with average concentrations derived from a macroscopically valid rate equation treatment of chemical kinetics, such as given by **Eqs [2]** and **[3]**. This disagreement originates from the fact that the rate equation approach is valid in the thermodynamic limit wherein molecular fluctuations are, to good approximation, proportional to Ω^1/2^ (Ω being the volume of the relevant compartment or the system size); but this assumption is too restrictive for microscopic fluctuations in general. However, it can be shown using the well-known linear noise approximation [16] that for larger system size, the average calculated from the master equation obeys the macroscopic law for zero, first, and second order chemical reactions [17], which covers the region of validity of **Eqs [2]** and **[3]**. At mesoscopic scales, quantitative disagreement is more pronounced, but a more careful analysis of the higher orders of the system size expansion of the master equation can produce effective rate equations that are valid for any system size [17]. Despite an assumption of “small” fluctuations used to justify the linear mass-action kinetics of **Eq [2]**, we found an increasing trend of mutual information for longer daisy chains simulated using the SSA for 2 to 128 source molecules [13], in qualitative agreement with predictions based on the rate equation approach.

We investigate the consequences of the kinetic mechanisms associated with the MA and EB models for transcriptional cascades of length *n* = 1,…,7 through use of the stochastic simulation algorithm (SSA) [16] implemented within the KaSim v4.0 engine [18–20]. These simulations approximate the solution of the relevant chemical master equation, and, therefore, avoid many assumptions associated with the usual rate equation treatment at the cost of computational complexity. Simulations were initialized at steady state with kinetic parameters *k* = 4, *k*_*d*_ = 1, *k*_+_ = 0.1/4820, and *k*_−_ = 0.1. The value of *k* was chosen to ensure that *k*≫*k*_*d*_, and *K*_*D*_ = 4820 ensures that TF proteins must typically make multiple binding attempts before a single transcription event occurs. We set 〈*R*_0,0_〉 = 100 and fixed *B*_*i*,0_ = 3 for all links in the cascade, which is both a reasonable estimate for the number of DNA promoter sites available to a TF within a cell, and a further guarantee that transcription initiation will be rate-limiting, regardless of the actual values of the rate constants *q*_*i*_. (Even if the transcriptional rate constants are large, the low number of binding sites coupled with the large dissociation constant will severely limit how frequently new proteins can be produced.) Unlike in our previous theoretical approach [21], wherein the unregulated lead TF was assumed not to fluctuate in concentration until the time point of interest, we employ the more realistic assumption that *R*_0_ obeys the following differential equation, which we also stochastically simulate:

$$\frac{dR_{0}}{dt}=\langle R_{0,0}\rangle -k_{d}R_{0}.$$ [6]

A common issue plaguing the application of information theory metrics such as mutual information is the sensitivity of their calculated values to the choice of the bin size used in histogramming the data [22–25]. This is only an issue, however, when the underlying random variables are formally continuous, and must therefore be discretized post hoc to estimate the differential entropy. Although we are simulating the behavior of chemical kinetics models with continuous concentrations, our simulations respect the underlying discreteness of the real biochemical processes. As such, when computing the mutual information between the initial and final TF copy numbers for an *n*-link cascade,

$$I(R_{n};R_{0})=\sum_{R_{n},R_{0}=0}^{\infty }p(R_{n},R_{0})\log_{2}\left[\frac{p(R_{n},R_{0})}{p(R_{n})p(R_{0})}\right],$$ [7]

the probability mass functions on the right-hand side of the above expression can be interpreted as histograms with separate bins for each possible number of protein molecules. Note that the joint probability *p*(*R*_*n*_, *R*_0_) will be a two-dimensional histogram constructed from the subset of all same-time pairs of *R*_*n*_ and *R*_0_ counts.

To sufficiently sample such a large number of bins, a large number of data points is required [26]. To determine just how high a sampling density we require to obtain consistent values of the mutual information in **Eq [7]**, we first simulated a pair of uncoupled transcription factors that both obey the kinetics of **Eq [6]**, and whose initial, steady-state copy counts were 100 and 400, respectively. Since the stochastic fluctuations in the number of molecules of these two TFs are, by construction, independent and identically distributed (iid), their mutual information should, in principle, be exactly zero. Any finite ensemble of instantiations of this system will approach a zero mutual information only asymptotically; to determine a sufficient sampling density to approximate this condition, we simulated this system for 50 time units (the inverse unit to that of the degradation rate constant), taking snapshots of its molecular composition at regular intervals. By choosing the size of this interval differently, we were able to compile datasets containing between 10^3^ and 10^6^ samples, and for each dataset of a given number of samples, we performed 100 replicate simulations.

**Fig 2:**
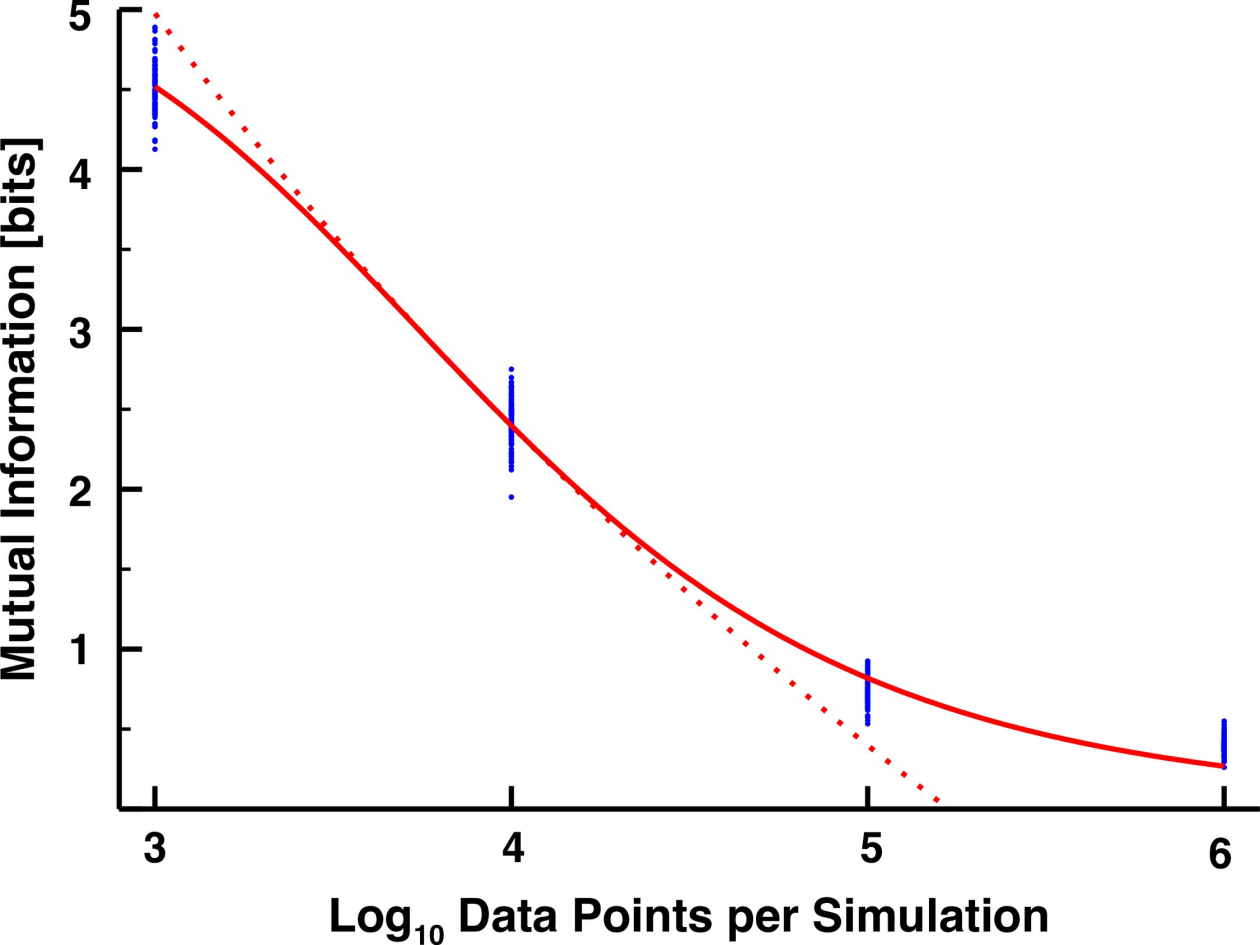
The mutual information (in bits) between two independent transcription factors as a function of the number of data points sampled per simulation. Different sampling densities were achieved by sampling the data more or less frequently, and 100 simulations were performed for each sampling frequency (blue dots). We fitted a sigmoidal relationship to the data (*y* = *y*_*max*_/(1+(*x*/*K*)^*h*^), *y*_*max*_ = 5.29832, *K* = 3.88923, *h* = 6.765666, solid red curve). We then approximated the power law region of the sigmoid with the line *y* = *m* ln *x*+*b*, *m* = −8.9617, *b* = 5.2269.

We plotted the mutual information values computed from each simulation in **Fig 2,** as a function of their sampling density. Although there is some expected variation across replicates, this pales in comparison to the variations across different sample sizes. Initially, with only 10^3^ data points per simulation, we calculated around 4.5 bits transferred, which is erroneously quite large, exemplifying the ability of spurious fluctuations to bias the value of the mutual information [27]. A three orders of magnitude increase in the number of data points is required to decrease this value to 0.25 bits, far below the 1-bit threshold required to determine with precision if the signal is above or below the mean. This monotonically decreasing trend of the mutual information toward zero with increasing sampling density is well fit with a sigmoidal equation, in which a line drawn from the slope at its inflection point crosses the log-scaled sample axis (*x*-axis) at approximately 5.23 (**Fig 2**, red dotted line). This suggests that we need at least 10^5.23^ ≈ 170,000 data points to sufficiently reduce the impact of an imperfect sampling methodology on the value of the calculated mutual information.

**Fig 3:**
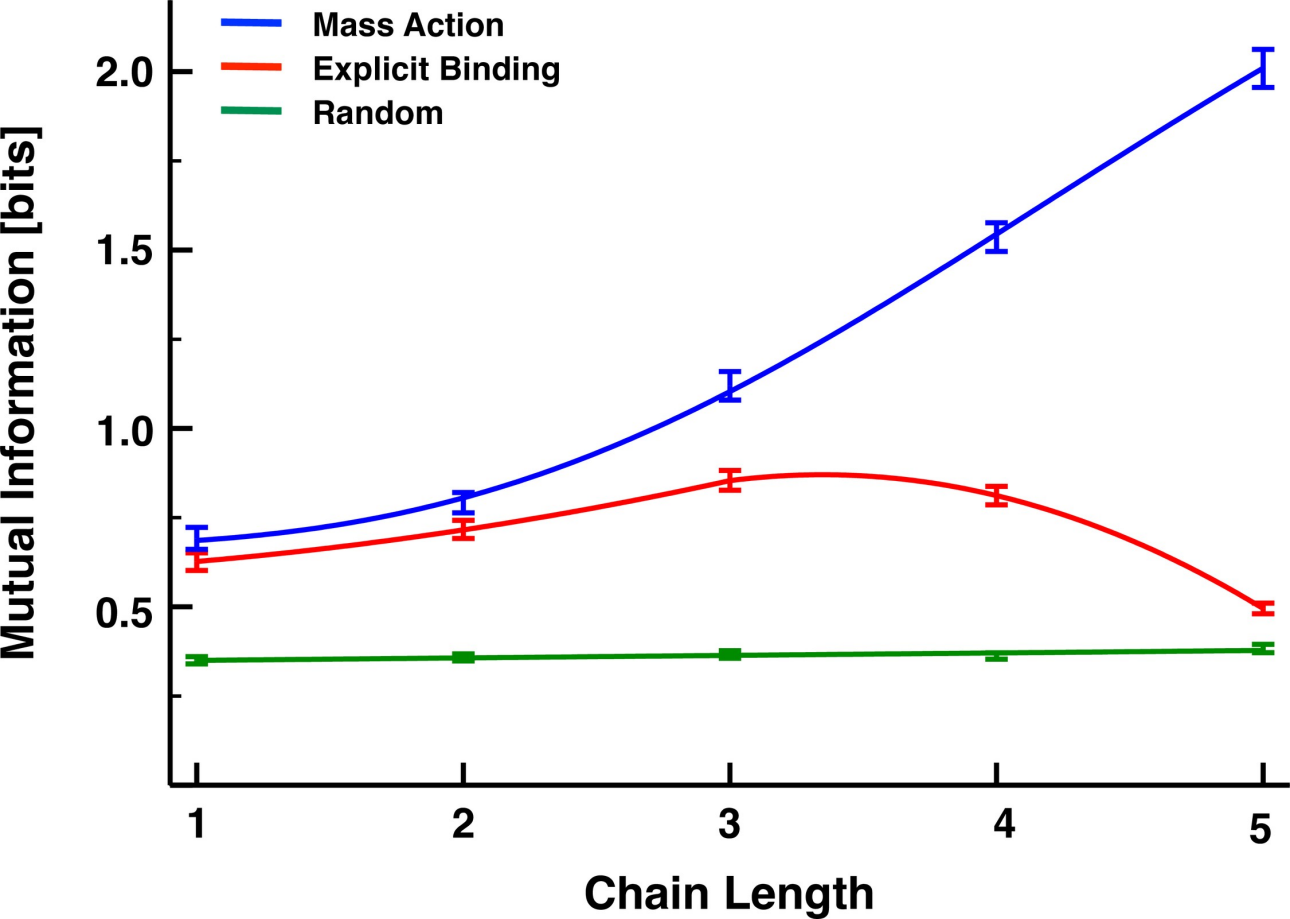
**Mutual information as a function of chain length for the MA (blue), EB (red), and NI (green) models.** This latter model, whose mutual information should be formally zero, provides a measure of the magnitude of the spurious correlations resulting from our sampling choices. The bars represent the 95% confidence intervals of the mean mutual information as measured by bootstrapping from 100 replicate simulations. The mass-action model results were fit with the sigmoid *y* = *y*_*min*_+*x*^*h*^(*y*_*min*_+*y*_*max*_)/(1+(*x*/*K*)^*h*^), *y*_*min*_ = 0.67027, *y*_*max*_ = 3.45238, *K* = 175.58899, *h* = 3.16516. The explicit-binding model results were fit piecewise with the quadratic *y* = *ax*^2^+*bx*+*c*; *a* = 0.02473, *b* = 0.01435, *c* = 0.58801 for *x*≤3, and *a* = −0.14725, *b* = 0.91883, *c* = 0.66762 for *x*>3.

Based on this analysis, we simulated each interacting transcriptional cascade for 50 time units, capturing snapshots of the total copy number of each molecular species every 5 x 10^−5^ time units. We then calculated the mutual information between *R*_0_ and *R*_*n*_ for each of 100 replicate simulations. These replicate-averaged results are now shown in **Fig 3** as a function of cascade length for the MA (blue), the EB model (red), and a model with non-interacting/uncorrelated gene products accumulated at the same steady-state concentrations (green). We term this latter model, the non-interacting (NI) model. The error bars represent 95% confidence intervals, which we obtained by bootstrapping the results of the simulations with replacement 1000 times. Although the mutual information also increases with chain length for the explicit-binding model, this trend plateaus for chains of approximately *n*≈3−4 links, and for longer chains, the mutual information rapidly decays toward the value given by the non-interacting model. Although biology should generally disfavor longer chains simply due to the greater metabolic burden they place on the cell, they also appear unfavorable from a signaling perspective.

**Fig 4:**
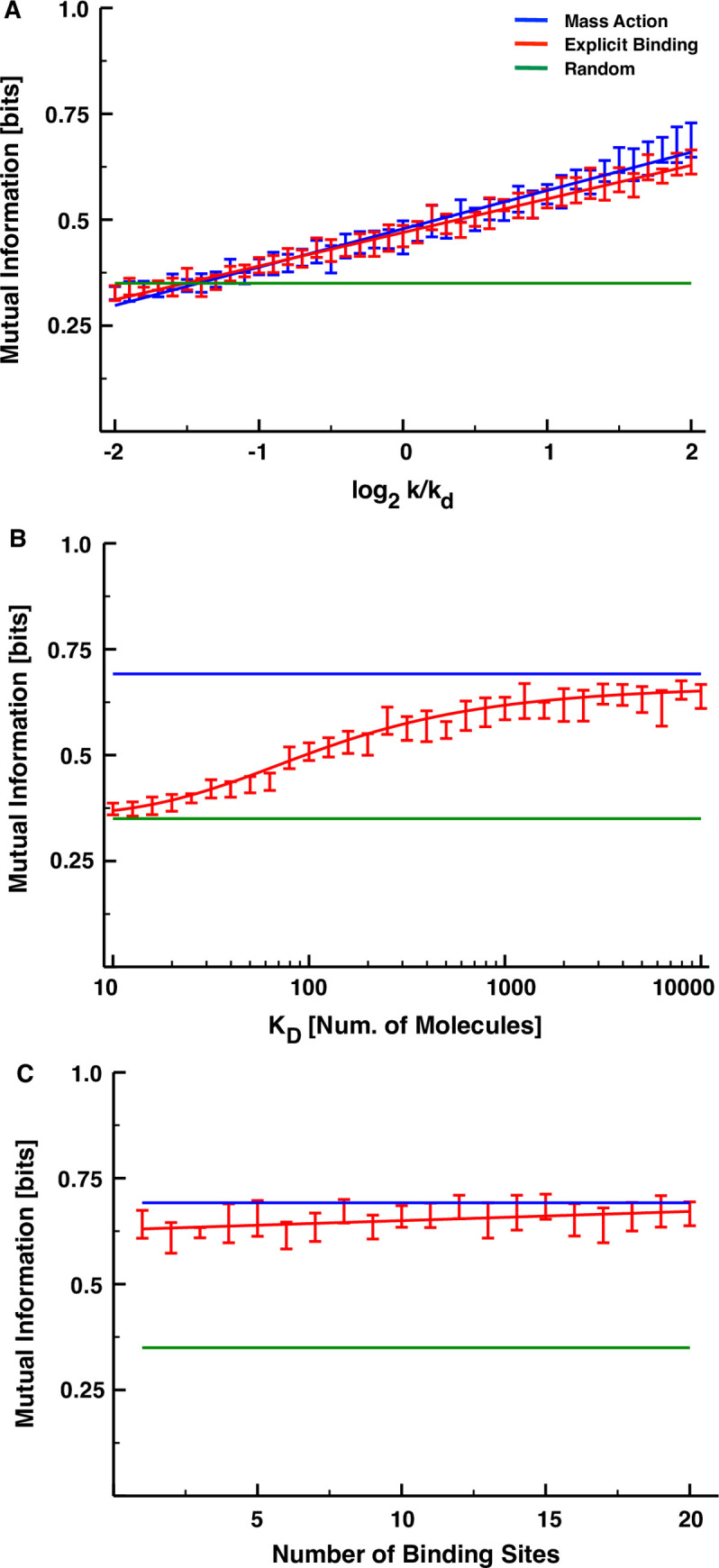
Sensitivity of the mutual information of each model on the parameters. (A) Mutual information as a function of *k/k*_*d*_ for the MA (blue) and EB models (red). The bars represent the estimated 95% confidence intervals of the mean mutual information as determined by bootstrapping the results of 100 replicate simulations. Each model was fit to the function y = ax + b, with a = 0.090467, b = 0.478574 for the mass-action model and a = 0.079252, b = 0.469904 for the explicit-binding model. (B) Mutual information as a function of *K*_*D*_ for the explicit-binding model. These results were fit with the sigmoid y = y_min_ + (y_max_−y_min_)(x^h^)/(K + x^h^), y_min_ = 0.356155, y_max_ = 0.667316, K = 22.917036, h = 4.367485. (C) Mutual information as a function of the number of binding sites for the explicit model. These results were fit with the line y = mx + b, with m = 0.0021724, b = 0.6280985.

The EB model is clearly less informationally efficient than the MA model for the set of parameters chosen in **Fig 3**, but we now demonstrate that the EB model is less efficient for any set of parameters. To prove this, we consider only a single regulatory interaction (*n* = 1) and show that none of the three control parameters of the explicit-binding model can make it outperform the mechanistically simpler alternative. The first parameter we consider is the ratio *k*/*k*_*d*_, which controls the steady-state ratio of concentrations 〈*R*_1,0_〉/〈*R*_0,0_〉, and, in **Fig 4A**, we plot the mutual information for both the explicit-binding and mass-action models for a single-link cascade as a function of this dimensionless parameter. We varied *k*/*k*_*d*_ from 2^−2^ to 2^2^, and for each value from within this interval, simulated the models exactly as before, while keeping all other parameters the same as those used to produce the curves of **Fig 3**. At any given value of *k*/*k*_*d*_, we find that the two models share a similar amount of information, and in both cases this information grows roughly linearly with the logarithm of the control parameter. The best-fit line to the MA simulation data does, however, exhibit a statistically significantly steeper slope than that of the EB model (see **Table 1**), which suggests that there could be statistically significant differences between the fluctuation sensitivities of the two models at much larger or much smaller values of *k*/*k*_*d*_. In the former case, this difference would favor the mass-action model even more, and in the latter case, the mutual information values would be indistinguishable from noise. (Recall from **Fig 3** that for datasets with a million samples, this indistinguishability threshold fell at roughly 1/4 of a bit.)

**Table 1:** Estimates of the parameters for the function *y* = *ax*+*bzx*+*c*+*dz*, fitted to the mean mutual information for increasing *k*/*k*_*d*_ values for the mass action model (z = 1) against the explicit binding (z = 0).

A	b	p-value	c	d	p-value
0.79252	0.011214	4.45e-5	0.469904	0.008671	0.00597

Significant p-values indicate that the values of b and d are relevant, meaning that the slope and intercept of the best-fit curves for the two models are significantly different.

The EB model has two control parameters that are not present in the MA or NI models: the dissociation constant *K*_*D*_ and the number of TF binding sites *B*_*i*,0_. In **Fig 4B**, we fix *k*/*k*_*d*_ and *B*_*i*,0_ to the values used in **Fig 3**, and plot the resulting mutual information as a function of *K*_*D*_ for a single-link cascade obeying explicit-binding kinetics. The dashed lines in the plot mark the mutual information levels for the mass-action and non-interacting models, and the sigmoid curve we use to fit the EB simulation data appears to saturate at the former for large *K*_*D*_ and the latter for small *K*_*D*_. In other words, this means that if the binding of transcription factors to DNA is too efficient, differing concentration fluctuations cannot be discriminated by the transcriptional mechanism; but no matter how inefficient the binding becomes, the fluctuation sensitivity can never surpass the mass-action limit. In **Fig 4C** we repeat this exercise varying *B*_*i*,0_ while keeping all other parameters fixed, and we see that there is only a very weak growth trend in the mutual information with the number of binding sites (slope = 0.0021724, p = 0.00432). If it is possible for the fluctuation sensitivity of the EB model to exceed that of the mass-action model for a sufficiently large number of binding sites, it would clearly have to be for a biologically infeasible number of them.

To better understand the trends observed in **Fig 4**, we analyze the EB model within the previously-developed linearized kinetics framework [14]. Starting from **Eq [3]** and applying the Briggs-Haldane QSSA for *R*_*i*_∙*B*_*i*_, we can reduce the 2*n* differential equations governing our *n*-link cascade to only *n* coupled equations:

$$\frac{dR_{i}}{dt}=\frac{q_{i}(B_{i,0})(R_{i-1})}{K_{D}+R_{i-1}}-k_{d}R_{i}.$$ [8]

Taylor expanding **Eq [8]** about steady state and keeping only terms of linear order, we then get the following:

$$\frac{d\delta R_{i}}{dt}={\tilde{k}}_{i}\delta R_{i-1}-k_{d}\delta R_{i}+\eta_{i},$$ [9]

wherein we have explicitly added the stochastic noise term and defined an effective rate constant ${\tilde{k}}_{i}$ as:

$${\tilde{k}}_{i}\equiv \frac{q_{i}B_{i-1,0}K_{D}}{{\left(K_{D}+\langle R_{i-1}\rangle \right)}^{2}}.$$ [10]

If we assume that 〈*R*_*i*,0_〉≈〈*R*_*i*_〉 when 〈*R*_*i*,0_〉≫*B*_*i*,0_, then **Eq [4]** can be used to express **Eq [10]** in terms of the mass-action rate constant *k* instead of *q*_*i*_:

$${\tilde{k}}_{i}=\frac{kK_{D}}{K_{D}+\langle R_{i-1}\rangle }.$$ [11]

The above set of linearized rate constants can then be substituted into our previously reported, approximate formula for *I*_∞_, which is the long-time limiting value of the mutual information transferred by an *n*-link signaling cascade whose kinetics consist of small concentration fluctuations about steady state (see **Eq [27]** of reference [13]):

$$I_{\infty }(n,\{{\tilde{k}}_{i}\})=\frac{1}{2}\log\left\{1+\frac{{\left(\prod_{i=1}^{n}{\tilde{k}}_{i}\right)}^{2}/k_{d}^{2n}}{\sum_{m=1}^{n}\prod_{j=1}^{m-1}\left[{\tilde{k}}_{n-(j-1)}^{2}/{\left(2k_{d}^{2}\right)}^{m-1}\right]}\right\}.$$ [12]

**Fig 5:**
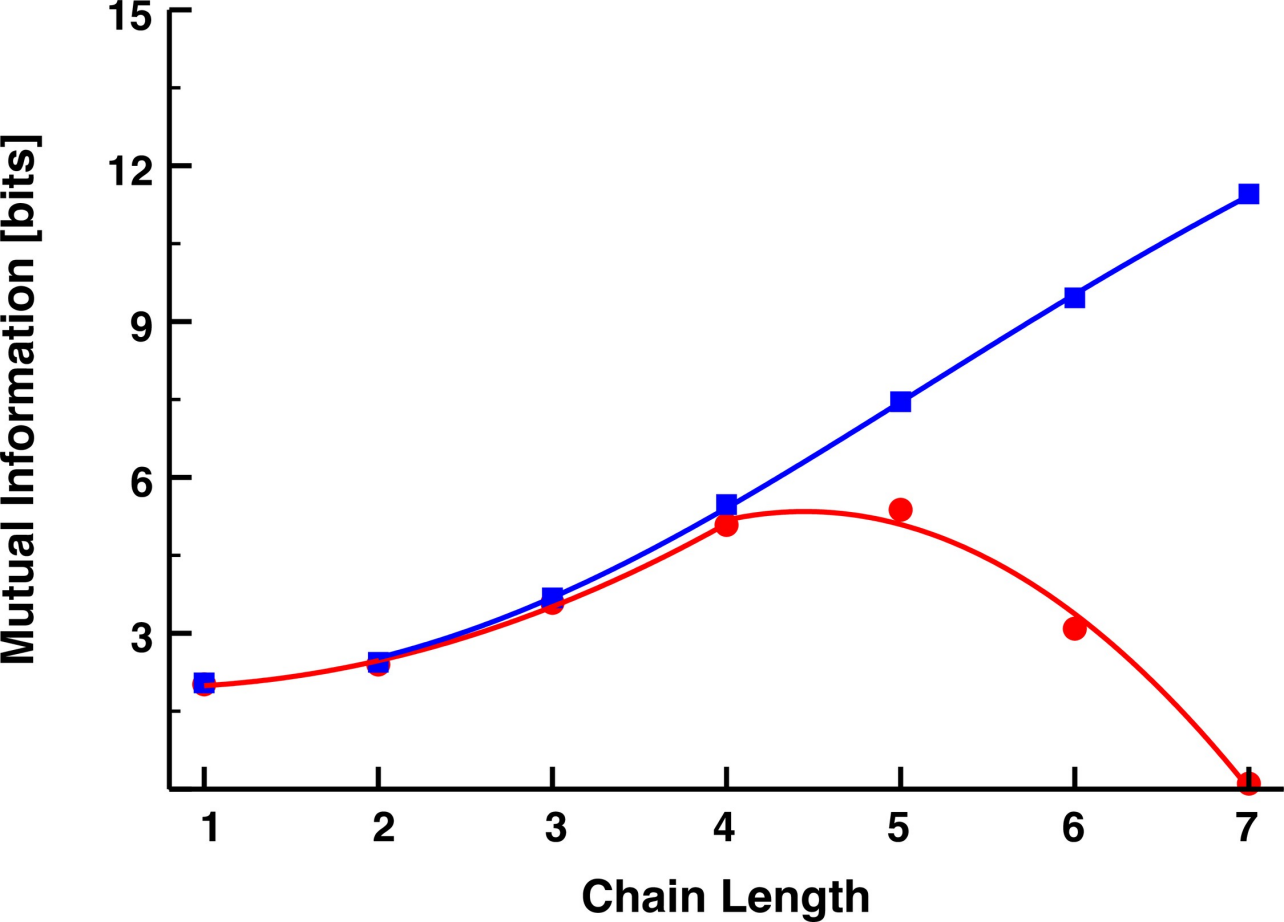
**Theoretical predictions of the mutual information (in bits) for a signaling cascade described by the EB model kinetics (red) and MA model kinetics (blue).** All parameters were chosen the same as those used in **Fig 3**, and the mutual information plots from that figure are replotted here for ease of comparison. The results from the MA simulations were fit with the sigmoidal function *y* = *y*_*min*_+*x*^*h*^(*y*_*max*_−*y*_*min*_)/(*K*+*x*^*h*^), *y*_*min*_ = 1.88496, *y*_*max*_ = 20.17665, *K* = 179.79606, *h* = 2.71205. The results from the EB simulations were fit piecewise with the quadratic *y* = *ax*^2^+*bx*+*c*; *a* = 0.27913, *b* = −0.35633, *c* = 2.06838 for *x*≤4; *a* = −0.8188, *b* = 7.2838, *c* = −10.8557 for *x*>4.74923792.

In **Fig 5**, we plot the mutual information of **Eq [12]** for both the explicit-binding and mass-action models as a function of cascade length, using the same parameter values from **Fig 3**. Although **Eq [12]** considerably overestimates the value of the mutual information for both models, it qualitatively captures the features of the simulation results: information associated with the EB model and its reversible binding mechanisms is bounded from above by the MA model results; and, after initially growing with the number of signaling links, it rapidly decreases towards zero. This theory also overestimates how many links it takes to reverse the growth trend in the mutual information (five rather than three), but this, along with the overall larger information values, can be attributed to our theoretical framework ignoring the noise in the signal, which would no doubt reduce the informational efficiency of the cascade.

Noting that the condition for the mutual information in **Eq [12]** to grow with *n* is roughly ${\tilde{k}}_{i}\gg k_{d}\forall i$, one can use **Eq [11]** to demonstrate that there is in fact no regime in which the EB model kinetics can achieve the monotonic growth in fluctuation sensitivity that is possible in the limit of the MA model. If on the one hand, we choose *k*>*k*_*d*_, then the steady-state concentration 〈*R*_*i*−1_〉 will grow monotonically with *i*, leading ${\tilde{k}}_{i}$ to invariably become smaller than *k*_*d*_ after some critical value of *i*. If, on the other hand, we choose *k*<*k*_*d*_, then the sequentially decreasing values of the steady-state concentrations will eventually reduce the effective rate constant in **Eq [11]** to approximately the value of *k*, which is less than *k*_*d*_ by assumption. We can also use the above equations to account for all of the trends observed in **Fig 4** by substituting **Eq [11]** into **Eq [12]** for the case *n* = 1:

$$I_{\infty }(1,{\tilde{k}}_{1})=\frac{1}{2}\log_{2}\left[1+{\left(\frac{kK_{D}}{k_{d}(K_{D}+\langle R_{0})}\right)}^{2}\right].$$ [13]

So long as the squared term inside the argument of the logarithm in **Eq [13]** is much larger than unity, the information clearly scales as log(*k*/*k*_*d*_). As *K*_*D*_→0, the information approaches zero, and as *K*_*D*_→∞, the information approaches the limit of the MA model, wherein ${\tilde{k}}_{1}=k$. Finally, the information in **Eq [13]** does not depend at all upon the number of binding sites, *B*_0,0_.

## Discussion

In this work, we set out to determine whether the ability of biological signaling cascades to sidestep the limitations of the data-processing inequality—a prediction made by a previously developed theory based upon a linearization of the fully nonlinear kinetic mechanism—was actually attainable in a model that did not rely on as many coarse approximations, and that explicitly accounted for certain aspects of the real biology of cellular transcriptional signaling. The EB model we employed, while still a gross simplification of real biology, at least required the transcription factors in charge of protein regulation to reversibly bind to DNA before being able to influence the rate of gene translation and subsequent transcription. By stochastically simulating the full nonlinear kinetics of this model, we were able to avoid making many of the approximations required to make the linearized kinetic theory algebraically tractable. Nonetheless, we still found that the information transmitted across a transcriptional signaling cascade can increase with the number of regulatory links—it just cannot grow indefinitely. After increasing for a few links, the signal abruptly becomes indistinguishable from noise after only an additional link or two. We found that our linearized theory, when applied to the EB model equations, can reproduce this phenomenology, though it grossly overestimates the absolute magnitude of the mutual information. This enabled us to justify our finding that simpler models free of reversible binding kinetics, which allow transcription factors to directly regulate protein synthesis without first binding to a DNA promoter site, provide an upper bound on the informational efficiency of the EB model, even for short cascades where both models predicted monotonic, link-by-link signal amplification. This result in particular suggests that the kinetics of protein binding serve as a sort of signal dampener that further complicates the evolutionary narrative of molecular communication in biological systems.

Due to the high number of different signaling molecules crisscrossing the cellular cytoplasm, protein binding requires a high level of specificity to be effective, and it must also be reversible, so that binding sites are not occupied longer than necessary. These constraints favor protein dissociation over association, which means that multiple cycles of binding and unbinding must typically occur before processes like transcription can successfully initiate. This results in a separation of time scales, wherein the kinetics of association and dissociation can be thought to exist at all times in a quasi-steady state with respect to the kinetics of transcription itself. When the steady-state protein concentrations grow across the length of a cascade (generally true when the rate of transcription outpaces that of protein catabolism), this quasi-steady fraction of occupied binding sites will approach saturation with each successive link. Once this saturation is reached, the number of bound proteins will, on the time scale of transcription, effectively not fluctuate. In this limit, a fluctuation in the number of free TF molecules (the signal) cannot be transmitted, since a commensurate fluctuation in the concentration of bound TF molecules cannot be induced. This essentially adiabatic regime is fundamentally why the fluctuation sensitivity of an explicit-binding cascade inevitably falls off after enough links: increasing the amount of transmitted information requires an amplification in the number of proteins, but this amplification saturates the rate of transcription, thereby rendering the kinetics insensitive to fluctuations.

In addition to limiting the length of regulatory cascades over which information can be meaningfully transmitted, a saturating rate of transcription also suppresses the absolute amount of information that can be transferred over a cascade below the single-bit threshold. Less than a single bit of information corresponds to a response of “maybe” to a “yes” or “no” question, suggesting that individual cells struggle with even a binary response to environmental changes. This low communication capacity is consistent with past investigations that have found an association between poor intracellular communication and efficient population level responses [28]. Cells typically exist as part of a large population, and adaptation to an environmental change seldom requires the participation of every single cell. Low fidelity communication within each individual cell ensures that only a fraction of the population will succeed in responding to a stimulus, and this can actually be healthier for the community as a whole by conserving resources and avoiding a population-amplified response that exceeds the scale of the triggering stimulus.

Our modeling of the effect of binding kinetics on information transmission along signaling chains is general enough to suggest a molecular role in constraining biological network structure. Gene regulatory networks, for example, may grow through an evolutionary mechanism that involves gene duplication and divergence to generate new regulatory interactions [29]. Although networks modeled statistically with this growth mechanism have some topological similarity with known gene-regulatory networks (i.e., they are “scale-free,” “small world” networks [30]), they do not explicitly account for the underlying regulatory mechanisms which connect network structure with function and phenotype [31]. Our information-theoretic analyses identify a signaling “length” scale for these and possibly other molecular networks, suggesting a new mechanism of consideration in models that hope to explain the large-scale structure of molecular networks. If the structure of these networks is constrained, in part, by molecular binding events, then our theory predicts that longer chains should exhibit binding interactions that are weaker (larger *K*_*D*_) than comparatively shorter chains. Experiments could test this hypothesis, for example, by comparing the value of curve-fitted rate constants for the kinetic activity of fluorescent protein reporters in shorter and longer regulatory chains of protein expression.

To determine whether such experiments are possible, we reviewed datasets from the BIOGRID database [32], which provides a number of gene regulatory networks obtained for singular and multicellular organisms. We reviewed datasets for Saccharomyces cerevisiae (baker’s yeast), the Escherichia coli bacterium, Drosophila melanogaster (fruit fly), Mus musculus (house mouse), and Homo sapiens, searching them for regulatory daisy chains of 3, 4, and 5 nodes with, respectively, 2, 3, and 4 links. Specifically, we searched for regulatory daisy chains in which none of the intermediate genes exhibited interactions beyond the adjacent ones. We found only the vertebrate datasets exhibited regulatory daisy chains with up to 3 links, and no datasets we reviewed had any chains with 4 links. For example, in the mouse dataset, we identified 2699 2-link and 148 3-link daisy chains. A more thorough analysis of the functions associated with these chains is beyond the scope of our discussion, but their existence shows that cell-based expression assays could be used as a basis to test the general results from our mathematical models.

Ultimately, we have demonstrated that seemingly small mechanistic details can have a profound impact on how information flows through a system. By better understanding how the granular mechanisms of molecular signaling events impact the communication capacities of complex biological networks, we can perhaps one day use mechanistic knowledge to make predictions about network topology or vice versa. For example, the lack of long, linear cascades in the transcriptional network of the bacterium *Escherichia coli* may in fact be nature’s attempt to compensate for the very limitations on information flow that we have predicted with our modeling.
